# Molecular Simulation Study of All-Silica Zeolites for the Adsorptive Removal of Airborne Chloroethenes

**DOI:** 10.1021/acs.langmuir.4c03947

**Published:** 2025-01-06

**Authors:** Michael Fischer

**Affiliations:** a Faculty of Geosciences, University of Bremen, Klagenfurter Straße 2-4, Bremen 28359, Germany; b Bremen Center for Computational Materials Science (BCCMS) and MAPEX Center for Materials and Processes, University of Bremen, Bremen 28359, Germany

## Abstract

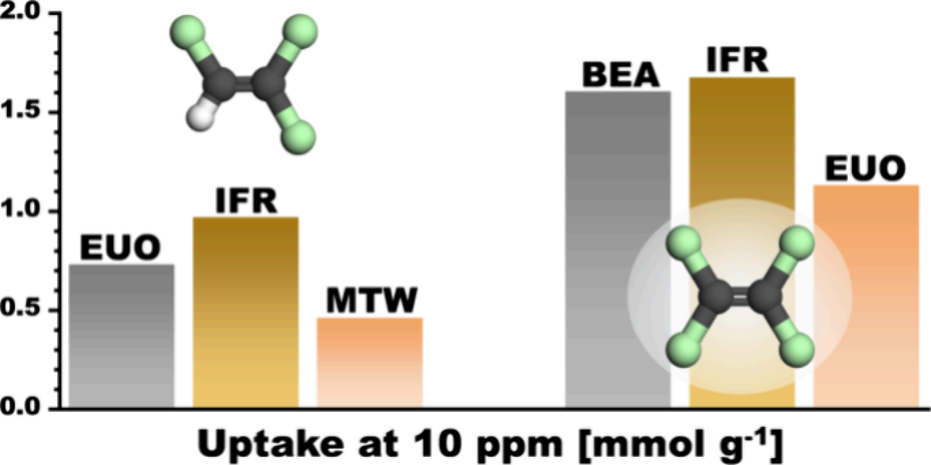

Chloroethenes (C_2_H_4–*x*_Cl_*x*_ with *x* = 1, 2, 3, 4) are produced and consumed in various industrial processes. As the release of these compounds into air, water, and soils can pose significant risks to human health and the environment, different techniques have been exploited to prevent or remediate chloroethene pollution. Although several previous experimental and computational studies investigated the removal of chloroethenes using zeolite adsorbents, their structural diversity in terms of pore size and pore topology has hardly been explored so far. In this work, molecular simulations using validated empirical force field parameters were used to study the gas-phase adsorption of chloroethenes in 16 structurally distinct zeolite frameworks. As all of these frameworks are synthetically accessible in high-silica form, the simulations used purely siliceous zeolite models. In the most relevant concentration range (0.1 to 10 ppm by volume), substantial uptakes of tri- and tetrachloroethene were computed for several zeolite frameworks, prominently EUO, IFR, MTW, MOR, and BEA. In contrast, vinyl chloride uptakes were always too low to be of practical relevance for adsorptive removal. For selected frameworks, simulation snapshots were analyzed to investigate the impact of pore shape and, at higher uptakes, guest–guest interactions on the adsorption behavior. Hence, this study not only identifies zeolites that should be prioritized in future investigations but also contributes to the microscopic understanding of chloroethene adsorption in crystalline microporous materials.

## Introduction

Chloroethenes (abbreviated CEs throughout this article) of the general formula C_2_H_4–*x*_Cl_*x*_ with *x* = 1, 2, 3, 4 are widely used in various industrial processes:^1^ The monosubstituted form, vinyl chloride (chloroethene), one of the world’s most important commodity chemicals, is primarily used as a monomer in the production of polyvinyl chloride (PVC), a widespread and versatile polymer. Among the three dichloroethene isomers, 1,1-dichloroethene also finds use as a monomer in the synthesis of polymers. The *cis* and *trans* forms of 1,2-dichloroethene, which occur as byproducts in some processes, are in limited demand, with niche applications as solvents and cleaning agents. In contrast, trichloroethene is rather widely used, primarily in the degreasing of metal parts and as an intermediate in the production of hydrofluorocarbons and other chemicals, with additional applications including industrial dry cleaning and the production of inks and paints. Finally, tetrachloroethene is applied in similar fields, notably in dry cleaning, as a degreasing agent, and as feedstock in industrial syntheses. IUPAC names, frequently used synonyms and abbreviations as well as selected thermophysical properties of CEs are compiled in Table 1.

**Table 1:** Names, Abbreviations, Sum Formulas, PubChem Compound Identifiers (CIDs),^2^ Boiling Points *T*_b_ (at Ambient Pressure), and Vapor Pressures *p*_vap_ (at 293 K) of Chlorinated Ethenes and, for comparison, Ethene^a^

IUPAC name	synonym(s)	abbreviation	sum formula	PubChem CID	*T*_b_ [K]	*p*_vap_ [kPa]
ethene	ethylene	ETH	C_2_H_4_	6325	169	4,090 (at 273 K)
1-chloroethene	vinyl chloride, vinyl chloride monomer	VC	C_2_H_3_Cl	6338	260	343
1,1-dichloroethene	vinylidene chloride	1,1-DCE	C_2_H_2_Cl_2_	6366	305	66
(*Z*)-1,2-dichloroethene	*cis*-1,2-dichloroethylene	*cis*-1,2-DCE	C_2_H_2_Cl_2_	643833	333	21.6
(*E*)-1,2-dichloroethene	*trans*-1,2-dichloroethylene	*trans*-1,2-DCE	C_2_H_2_Cl_2_	638186	321	36.1
1,1,2-trichloroethene	trichloroethylene	TCE	C_2_HCl_3_	6575	360	7.76
1,1,2,2-tetrachloroethene	tetrachloroethylene, perchloroethylene	PCE	C_2_Cl_4_	31373	394	1.94

a Thermophysical properties were taken from the GESTIS property database.^3^

It has been established that various health hazards are associated with airborne CEs, even at relatively low levels. In addition to acute toxicity at high concentrations, long-term exposure may have negative health consequences. For example, VC has been reported to cause liver damage, nerve damage, and immunological dysfunction; moreover, it is known to be a human carcinogen.^3,4^ Whereas data regarding toxicity and mutagenicity/carcinogenicity of DCE isomers are relatively sparse,^5,6^ it is established that TCE can cause both acute and chronic toxic effects and it has also been classified as carcinogenic.^7,8^ For PCE, neurotoxic effects caused by long-term exposure are most concerning, and carcinogenic effects are suspected.^3,9^ Due to these negative health impacts, limits for workplace exposure to airborne CEs have been introduced in many countries. For example, Directive 2019/130 of the European Union sets the acceptable long-term exposure limits (reference time frame: 8 h) for VC and TCE to 1 and 10 ppm (ppm) by volume, respectively.^10^ In Australia, long-term exposure limits are 5 ppm for VC and 1,1-DCE, 10 ppm for TCE, 50 ppm for PCE, and 200 ppm for 1,2-DCE.^11^

Indoor air pollution by CEs is of most significance in facilities where these compounds are produced and/or used (*e.g.*, production plants, dry cleaners, etc.), although outgassing of CE-containing products may also play a role. Additionally, the pollution of different environmental compartments by CEs has raised concern with regard to potential negative effects on human health and the environment. The main sources of VC in the environment are emissions and effluents from plastic industries, with hazardous waste sites and natural gas extraction sites making additional contributions.^4^ In addition to these point sources, there have been a number of large-scale VC spills,^6^ the most notorious recent example being the derailment of a freight train transporting VC, among other hazardous chemicals, in East Palestine, Ohio (USA) in February 2023.^12^ Due to its high vapor pressure, most of the VC released to water or soils will volatilize quickly. In air, it degrades photochemically within a few days.^4^ While being of less concern due to their smaller production volumes, the DCE isomers have release pathways and environmental behavior similar to VC, with reductive dehalogenation of TCE and PCE to (mostly) *cis*-1,2-DCE being an additional source of DCE in the environment.^5,6^ TCE is primarily released into the atmosphere by evaporation from degreasing operations and other industrial uses, resulting in higher atmospheric TCE levels in industrial areas as compared to rural regions.^7,8^ Although it mostly volatilizes from water and soils, it may also enter subsurface areas where it is relatively persistent. The most significant release of PCE arises from fugitive emissions of the dry cleaning industry, with emissions related to degreasing and other solvent uses also playing a role.^9^ Due to its long atmospheric half-life of several months, PCE has been detected in remote locations, far away from emission sources. Disposal of PCE-contaminated sludges and filters contributes to the pollution of soil and groundwater, where it is very persistent. Both TCE and PCE have been classified as “emerging outdoor airborne pollutants” in a recent survey that aimed to identify pollutants of particular importance for better monitoring and future policy development.^13^ Among a total of 262 emerging pollutants, TCE and PCE were proposed for the second-highest prioritization category, behind only acrylonitrile and 1,3-butadiene (and having the same priority as toluene and arsenic).

In industrial processes, gaseous emissions of CEs are typically collected and incinerated, and air- or steam-stripping is used to separate CEs from industrial wastewaters prior to incineration of the gas.^1^ For facilities where incineration is not possible, removal processes using adsorption, absorption, or condensation have been proposed, and a number of patents have been awarded in this area for VC removal in particular (see ref 471 in ref (1) for an overview). Regarding adsorption-based processes, activated carbons (ACs) and other carbonaceous materials have been widely characterized as suitable adsorbents for the removal of CEs from the gas phase^14−21^ and from aqueous solution.^22−30^

Zeolites, crystalline microporous materials having a tetrahedral framework structure, have been investigated altogether less frequently than carbons. Nevertheless, a number of experimental studies have addressed the adsorption of TCE and PCE using zeolites. Specifically, Giaya et al. compared Silicalite-1, an all-silica zeolite having the MFI framework type,^31^ a dealuminated zeolite Y (FAU framework), and an AC in terms of their TCE and PCE adsorption behavior, both for the gas phase and for aqueous solution.^32,33^ In addition to observing high uptakes of both species in Silicalite-1 in the ppm range, they also demonstrated that coadsorption of water affected CE adsorption in the all-silica zeolite much less than in the other two adsorbents, a finding that was attributed to its high hydrophobicity. In an investigation focused on cationic zeolites, Na-containing FAU-type zeolites with low Si/Al ratios (1.2 and 2.4) were found to be efficient adsorbents for the removal of PCE from dry gas streams.^34^ However, it was also noted that humidity would have to be removed prior to PCE adsorption as water adsorption had a detrimental effect on the adsorbent performance. Besides adsorptive removal, it was proposed to employ zeolites as catalysts for the oxidative destruction of CEs in the gas phase.^35−37^

A number of other investigations looked at gas-phase CE adsorption in zeolites from a more fundamental point of view.^38−47^ In particular, Mellot et al. and Chihara and co-workers investigated the adsorption of TCE and PCE (along with other chlorinated hydrocarbons) in FAU samples having different Si/Al ratios and obtained via different dealumination routes.^39,42^ The experimental investigations were complemented by molecular simulations (see below). The impact of relative humidity on TCE and PCE adsorption capacities of FAU-type zeolites was also studied.^41,44,45^ Although it was generally observed that more hydrophobic zeolites (higher Si/Al ratios) possess higher uptake capacities, increasing diffusion limitations in highly siliceous samples were highlighted in one of the studies. Detailed investigations revealed a rather unusual adsorption behavior of PCE in MFI-type zeolites, with the adsorption isotherm showing two steps.^40^ Different researchers have attributed this phenomenon to the adsorption of PCE in distinct regions of the pore^40,43^ and to structural transitions of the framework with increasing pressure.^46,47^

The ability of zeolite adsorbents to remove CEs from aqueous solution has received considerable attention.^48−52^ To limit the coadsorption of water, these investigations typically focused on highly siliceous zeolites. For example, Silicalite-1 membranes grown on steel supports were proposed for the removal of *trans*-1,2-DCE and TCE from water.^50,51^ Other authors used zeolites as “dual-function” materials that allow for a catalytic decomposition of the CEs after adsorption.^49^ In a very recent pilot-scale study by Georgi and co-workers, an Fe-loaded MFI-type zeolite was used together with an AC adsorber for the removal of hydrophilic chlorinated hydrocarbons including VC from contaminated groundwater.^52^ The zeolite could be regenerated several times by flushing with H_2_O_2_, degrading the adsorbed contaminants in a Fenton-like reaction. Since the zeolite removed those compounds that break through the AC early, the operation time of the AC adsorber could be expanded significantly. This last example shows that zeolites may not necessarily be attractive as a stand-alone solution for contaminant removal, not least due to their high cost, but that usage in conjunction with other materials or techniques may constitute an attractive option.

In comparison with experimental studies of CE adsorption in zeolites, the number of investigations employing atomistic simulations is relatively limited. Complementing their experiments, Mellot and co-workers used Monte Carlo (MC) simulations with empirical force field parameters to investigate the role of guest–guest interactions between coadsorbed TCE molecules on the heat of adsorption in FAU-type zeolites.^39^ In subsequent work, they employed different models for zeolite Y, comparing a defect-free model to models containing an acid site (framework proton) and a silanol nest, respectively.^42^ For the more silica-rich composition studied (Si/Al ratio = 70), the computed heats of TCE and PCE adsorption were almost independent of the presence of acid sites or silanol nests, differing by not more than 2 kJ mol^–1^. Adsorption isotherms were calculated using MC simulations in the grand-canonical ensemble (GCMC), showing satisfactory agreement with experiment. Ahunbay^53^ and Jeffroy et al.^46,47^ employed MC simulations to rationalize steps in the PCE adsorption isotherms obtained for MFI-type adsorbents, with the former author also using molecular dynamics (MD) simulations to predict TCE and PCE diffusion coefficients. The role of Si/Al ratio and of water coadsorption on TCE adsorption was investigated, again for the case of MFI-type zeolites, by means of GCMC simulations.^54^ As in earlier studies, it was observed that hydrophilic areas (acid sites) in the zeolite structure have only a limited impact on the affinity toward TCE, while reducing the available pore space due to the preferred adsorption of water in these regions. A computationally efficient approach based on the calculation of Henry’s law constants (dubbed “Henry constants” in the following) through random insertion was pursued by Yazaydin and Thompson.^55^ These authors compared the affinities of four zeolites (MFI, BEA, FAU, MOR) to 1,1-DCE, proposing BEA as the most promising adsorbent for 1,1-DCE removal due to its high Henry constant (= high affinity).

Although a large number of topologically distinct framework types are available in high-silica or even all-silica forms, the structural diversity of zeolites in terms of pore size and pore topology has hardly been explored in the context of CE adsorption. The present study aims to fill this gap, employing a combination of Henry constant simulations and GCMC simulations to study the adsorption of all species listed in Table 1 in 16 all-silica zeolites. These particular frameworks were selected for the following reasons: (1) They can be synthesized in pure-silica or high-silica form.^31,56^ (2) They possess different pore sizes and connectivities. (3) According to preliminary simulations, described below, their pore apertures are large enough to permit the diffusion of all CE molecules. Through GCMC simulations for very low partial pressures, conclusions regarding the potential suitability of zeolites to remove trace amounts (down to subppm range) of CEs are drawn. Moreover, a closer look is taken at simulation snapshots in order to elucidate the microscopic origins of the observed affinities of the selected frameworks.

## Materials and Methods

### Models of Zeolites and Guest Molecules and Force Field Parameters

Throughout this work, zeolites are designated solely by their framework type code (FTC).^31^ The 16 frameworks that are in the focus of this study are listed in Table 2, grouped according to the size of the pore apertures. Four of the frameworks have channel systems lined by 10-membered rings of SiO_4_ tetrahedra (10MR), eight have 12MR pore openings, and two frameworks have pore systems consisting of both 12MR and 10MR channels. Finally, CFI and DON are “extra-large-pore” zeolites with 14MR channels. In addition to these 16 frameworks, the CHA framework was included in validation calculations for N_2_ and ETH. As this zeolite has 8MR pore openings, larger CEs like PCE cannot diffuse through the pore windows. Preliminary simulations of CE adsorption were performed for three other zeolites having 10MR windows, FER, MEL, and MWW. Unlike for the 16 zeolites listed in Table 2, interaction energy maps derived from the calculations indicated high energy barriers to diffusion of PCE, the heaviest CE, through the pore openings of these three zeolites. For this reason, FER, MEL, and MWW were not considered further.

**Table 2:** Information on Zeolite Pore Systems^a^

FTC	pore system and connectivity	*d*(LIS) [Å]	*d*(LDS) [Å]	*V*_acc_ [%]
MFI	10MR, 3D	6.4	4.7	9.8
MTT	10MR, 1D	6.2	5.1	8.0
TON	10MR, 1D	5.7	5.1	8.0
TUN	10MR, 3D	8.4	5.4	12.9
AFI	12MR, 1D	8.3	7.4	14.1
BEA	12MR, 3D	6.6	5.9	20.1
EUO	12MR, 1D	7.0	5.0	12.2
FAU	12MR, 3D	11.2	7.4	27.4
IFR	12MR, 1D	7.2	6.4	15.5
MEI	12MR, 3D	8.1	6.9	21.1
MOR	12MR, 1D	6.7	6.5	12.3
MTW	12MR, 1D	6.1	5.7	9.4
IWR	10 + 12MR, 3D	7.5	5.9	19.4
MSE	10 + 12MR, 3D	7.1	6.6	16.4
CFI	14MR, 1D	7.5	7.3	13.4
DON	14MR, 1D	8.8	8.1	15.6

a For each framework type, the dimensionality of the pore system (for pores ≥10MRs), the diameter of the largest included and largest diffusing spheres, and the percentage of accessible volume are given. All data were taken from the IZA database.^31^

All zeolite models were optimized using the GULP code^57^ and potential parameters developed by Sanders, Leslie, and Catlow (SLC).^58^ The majority of the SLC-optimized zeolite models were already used in a previous study, where information on the experimental structure data taken as starting points for the GULP calculations is provided.^59^ The newly optimized frameworks are listed in the following, including labels of type materials and references to experimental structure data: MTT (ZSM-23^60^), TUN (TNU-9^61^), EUO (EU-1^62^), MEI (ZSM-18^63^), IWR (ITQ-24^64^), and MSE (MCM-68^65^) as well as CHA.^66^ The optimized cell parameters are provided in the Supporting Information together with the size of the supercells used in Henry constant and adsorption isotherm simulations. CIF files of the SLC-optimized zeolite models are also provided as Supporting Information.

The structures of ETH and all CEs were optimized using parameters of the PCFF force field,^67,68^ and the N_2_ molecule was optimized using COMPASS parameters (although this force field is proprietary, parameters for certain small molecules are freely available^69,70^). The Lennard-Jones (LJ) parameters and partial charges from these force fields were also used to represent these molecules in the adsorption simulations. For the framework, two different sets of LJ parameters and charges were compared, namely, (1) the original PCFF parameters and (2) the potential parameters proposed by Emami et al. for use with PCFF (labeled PCFF/Emami throughout this work).^71^ As is standard for PCFF and COMPASS, a 9–6 form of the Lennard-Jones potential was used, and sixth-power combination rules were employed to compute parameters representing interactions between nonidentical atom types.^72^ PCFF parameters have been used in previous studies addressing the adsorption and diffusion of CEs in zeolites.^54,73^ Charges and LJ parameters are compiled in Table SII.

### Henry Constant Simulations

Henry constant simulations employed the Monte Carlo (MC) method in the uniform ensemble, in which single guest molecules are inserted at random, and the total energies obtained for a sufficiently large ensemble of configurations are used to compute the Henry constant *K*_H_.^74^ The selectivity toward a binary mixture of two species A and B in the limit of infinite dilution (zero coverage) can be calculated from the Henry constants as^75^
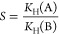


The isosteric heat of adsorption *q*_st_ in the limit of zero coverage can also be derived from these simulations as detailed by June et al.^74^

All Henry constant calculations were done for a temperature of 298 K using the *Sorption* module of the DS BIOVIA *Materials Studio* suite. The simulations used 25 million insertion steps, after ensuring that a further increase of the number of steps (up to 100 million steps) resulted in no significant change in the Henry constants. Using partial charges and LJ parameters as described above, Coulomb interactions were computed using the Ewald & charge group summation method with a cutoff of 18.5 Å, and van der Waals (vdW) interactions were evaluated using a pairwise summation, employing a cutoff of 18.5 Å and a spline-based truncation with a spline width of 1 Å.

### Grand-Canonical Monte Carlo Simulations

Monte Carlo simulations in the grand-canonical (μ*VT*) ensemble (GCMC), which also used the *Sorption* module, were performed to calculate single-component adsorption isotherms for ETH and all CEs in a fugacity range from 10^–5^ to 1 kPa (*T* = 298 K).

In addition to delivering the average number of particles per simulation cell ⟨*N*⟩ for given conditions, these simulations also allow calculation of the isosteric heat of adsorption according to^76^
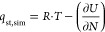


In this equation, which assumes ideal gas behavior of the fluid phase, *R* represents the ideal gas constant and the term in brackets corresponds to the change in total energy *U* upon change of the number of particles. As both host–guest and guest–guest interactions contribute to *U*, the heat of adsorption is loading-dependent.

These simulations used 10 million equilibration steps and 20 million production steps. Moreover, simulations of binary mixture adsorption were performed for VC/N_2_, PCE/N_2_, and TCE/N_2_ mixtures, assuming the same fugacity range for the CEs and fixing the fugacity of N_2_ to 101.325 kPa (*T* = 298 K). The number of equilibration and production steps was doubled in simulations of mixture adsorption. In all GCMC simulations, the probabilities of different types of MC moves were set to 2:1:1:0.1 for exchange (insertion/deletion):translation:rotation:regrowth moves, and the amplitudes of translation and rotation moves were rescaled to arrive at an acceptance probability of 0.5. To generate snapshots for adsorption simulations, MC simulations in the canonical ensemble (*NVT*) were performed for relevant loadings of the guest molecules of interest using otherwise analogous settings as in the GCMC simulations.

Charges, LJ parameters, and summation methods were the same as those described above in the Henry constant calculations. However, the cutoff distances were reduced to 12.5 Å to avoid the necessity to use very large supercells (the size of the supercells used in both types of simulations is compiled in Table SI of the Supporting Information). It was verified that a reduction of the cutoffs had only a small impact on the heat of adsorption, with changes in *q*_st_ on the order of one to four percent.

In all Henry constant and MC simulations, zeolite framework atoms were held fixed, and guest molecules were treated as rigid. To enhance the efficiency of the simulations, the insertion of the guest molecules was limited to the accessible part of the zeolite pores. The *Create Segregates* tool of *Materials Studio* was used to create a volumetric representation of the accessible part of the pore space.

## Results and Discussion

### Force Field Validation

**Figure 1:**
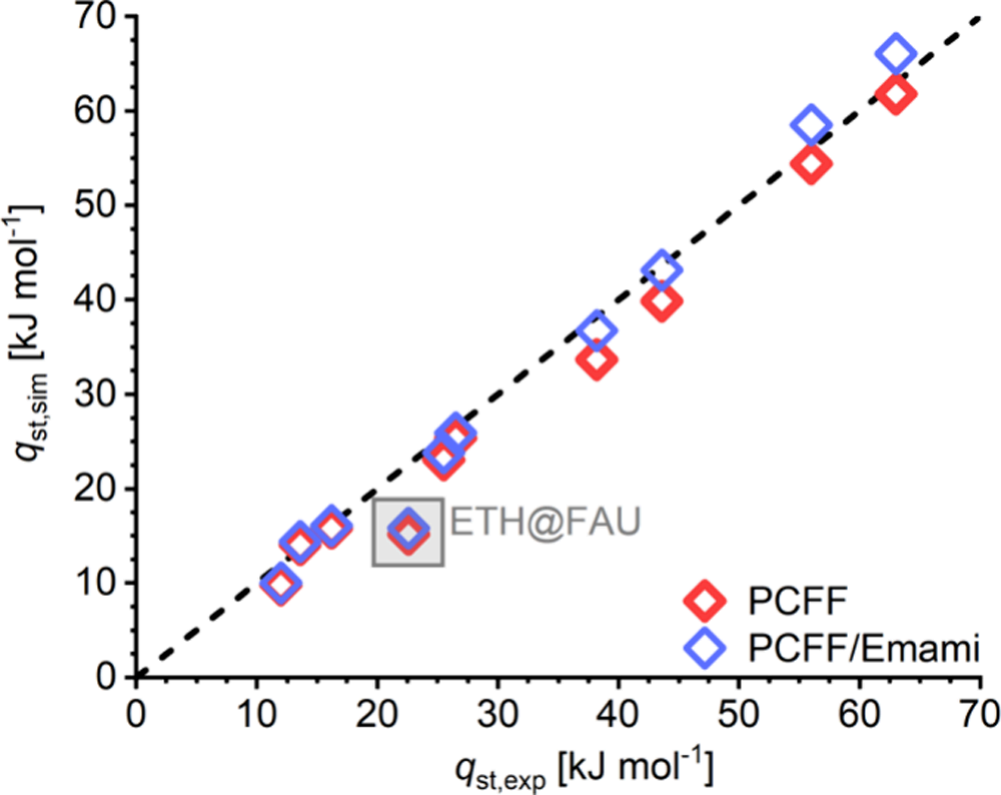
Comparison of the simulated heat of adsorption to experimental data. Experimental values are from the following sources (in order of increasing *q*_st,exp_): N_2_@FAU: ref (77), N_2_@CHA and N_2_@MFI: ref (78), ETH@FAU: ref (38), ETH@CHA: ref (79), ETH@MFI: ref (80), TCE@FAU and PCE@FAU: ref (42), TCE@MFI: ref (40), PCE@MFI: ref (47). For CHA and MFI, purely siliceous samples were studied, whereas investigations on FAU-type systems typically involved highly dealuminated zeolite Y samples.

Figure 1 compares the isosteric heats of adsorption *q*_st_ obtained from Henry constant calculations using the original PCFF parameters and PCFF/Emami parameters to the experimental values. The comparison covers N_2_, ETH, TCE, and PCE in FAU and MFI as well as N_2_ and ETH in all-silica CHA. As Figure 1 shows, agreement between simulation and experiment is good to excellent for 9 out of 10 data points, with the outlier being ETH@FAU (gray box). In this case, the calculated heats of ethene adsorption are about 30% lower than the experimental value. A potential origin of this discrepancy may be the use of a FAU-type sample dealuminated under harsh conditions in the experimental study, which is likely to contain a rather large amount of defects that might act as preferential adsorption sites for ETH.^38^ Since agreement between experiment and simulation is considerably better for all other cases, it seems reasonable to discard this data point. A calculation of the overall error for PCFF, using the remaining data points, delivers a mean of signed errors (MSE) of −1.9 kJ/mol and a mean of unsigned errors (MUE) of 2.0 kJ/mol. For PCFF/Emami, the MSE amounts to 0.0 kJ/mol and the MUE amounts to 1.4 kJ/mol. Although the difference in MUE is not large, the MSE is significantly reduced when using PCFF/Emami parameters, indicating that unlike for PCFF, there is no systematic underestimation of the heats of adsorption. In the view of the very good agreement, all calculations reported in the following used PCFF parameters for ethene and chlorinated ethenes, COMPASS parameters for N_2_, and parameters proposed by Emami et al. for Si and O atoms.

### Henry Constants and Heats of Adsorption at Zero Coverage

**Figure 2:**
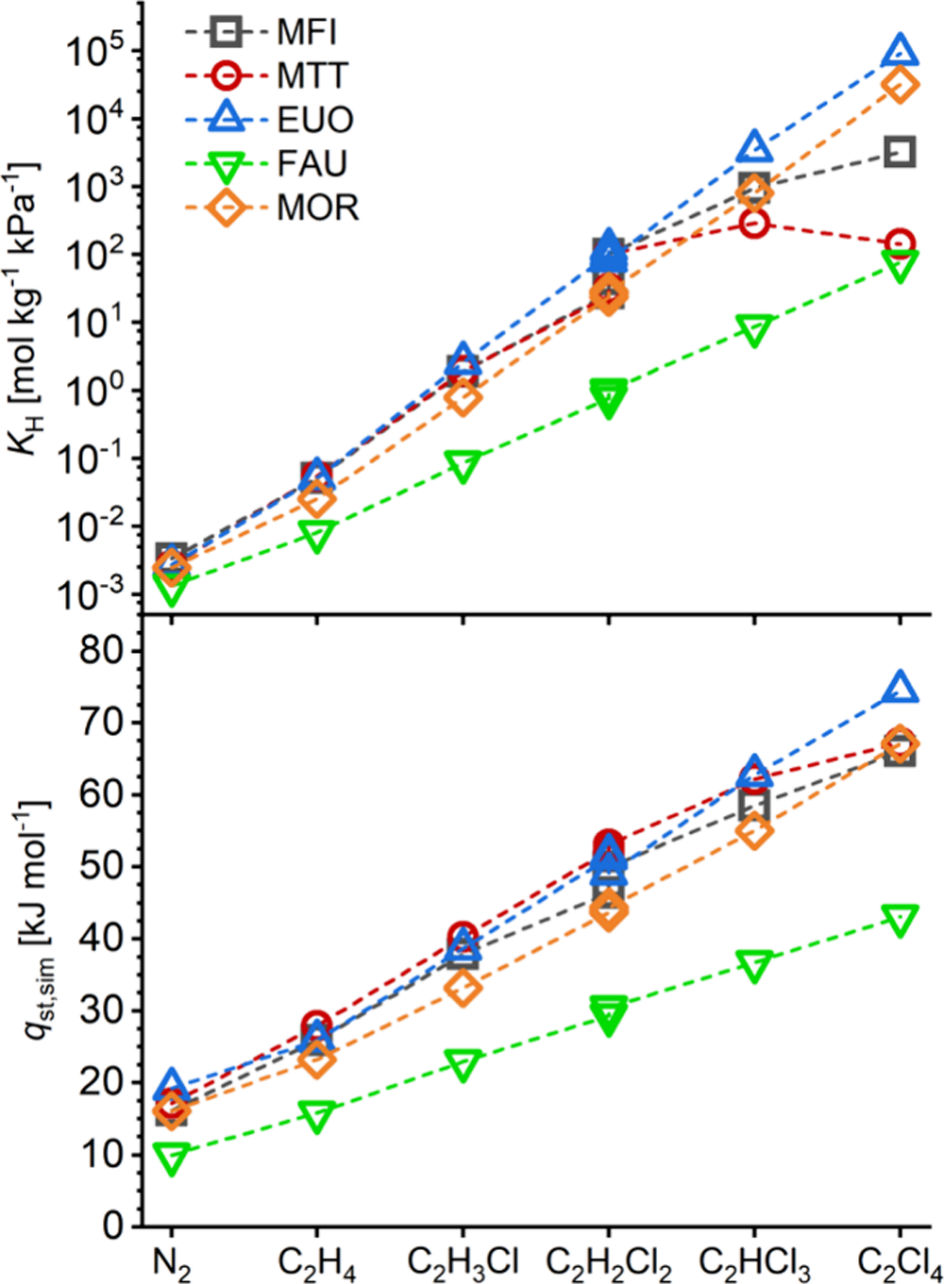
Calculated Henry constants (top, logarithmic scale) and isosteric heats of adsorption (bottom) for the five zeolites. Data points for all three DCE isomers are shown.

The full results of the Henry constant calculations are compiled in the Supporting Information (Table S1.1). Figure 2 illustrates typical trends in Henry constant *K*_H_ (shown on a logarithmic scale) and in the heat of adsorption *q*_st,sim_ for five zeolites, two with 10MR pore systems (MFI and MTT) and three having 12MR pores (EUO, FAU, and MOR). In addition to plotting the evolution for CH_4–*x*_Cl_*x*_ species as a function of the number of Cl atoms, the results for N_2_ are also included. It is clearly visible that the affinity toward all hydrocarbons is higher than for N_2_ and that the interaction strength increases with increasing number of Cl atoms, which is straightforwardly explained with the contribution of the polarizable Cl atoms to attractive vdW interactions. Among the different zeolites, the variation in affinity also increases: While the *K*_H_ values for ETH span only 1 order of magnitude, they extend over 3 orders of magnitude for PCE. The *K*_H_ values for N_2_ fall close together, indicating that the Henry’s law selectivities for heavier CEs over N_2_ will vary widely, as will be shown in more detail in the following subsection. For most zeolite–guest combinations, a close correlation between ln(*K*_H_) and *q*_st_ is apparent. An exception is visible for the heaviest CEs adsorbed in MTT, where the isosteric heat still increases when moving from TCE to PCE, but the Henry constant decreases. This can be attributed to the relatively narrow channels of MTT, for which the fraction that is accessible to TCE is significantly larger than the fraction that can accommodate the more voluminous PCE molecule.

### Henry’s Law Selectivities

**Figure 3:**
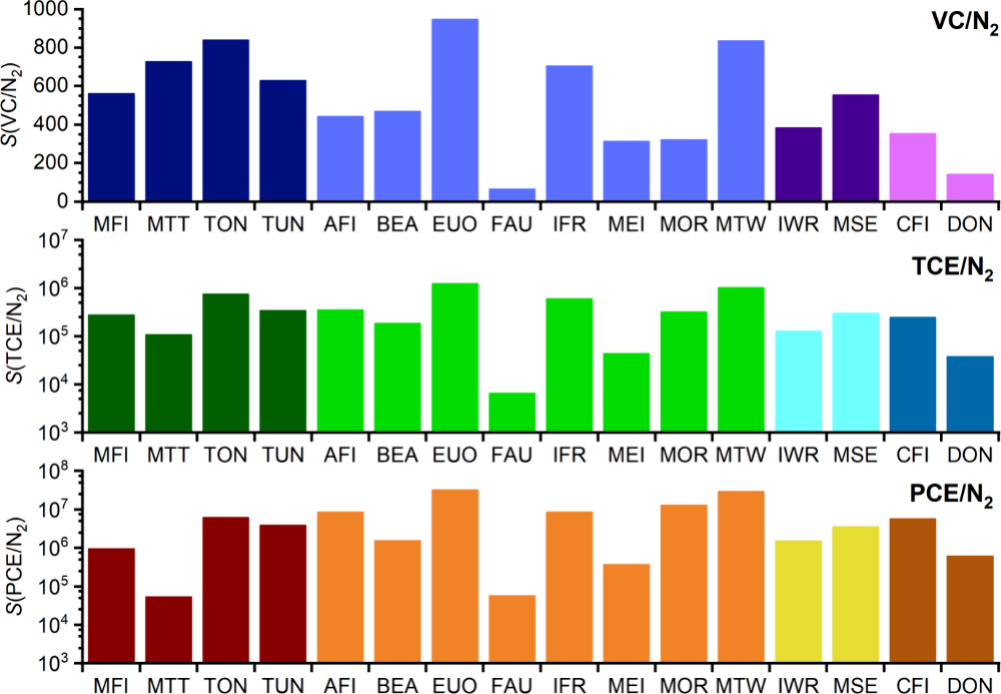
Henry’s law selectivities for VC (top), TCE (middle), and PCE (bottom) over N_2_. A logarithmic scale is used for TCE and PCE. From left to right, different colors are used to represent zeolites with 10MR, 12MR, 10 + 12MR, and 14MR pore openings.

Table S1.2a–c contains Henry’s law selectivities for ETH and CEs over N_2_, CEs over ETH, and *trans*-1,2-DCE over the other DCE isomers, respectively. Focus in this discussion will be placed on the selectivities for VC, TCE, and PCE over N_2_, which are compiled in Figure 3. For VC, a number of zeolites show selectivities on the order of 500 to 950, with EUO and TON being the most selective. Among the four zeolites that are commercially available in the high-silica form (MFI, BEA, FAU, and MOR), MFI exhibits the highest selectivity. For TCE and PCE, variations are much more pronounced with the computed selectivities spanning several orders of magnitude. TON, EUO, and MTW are most selective for TCE, with *S*(TCE/N_2_) on the order of 10^6^, whereas MOR and MFI are the best commercially available zeolites with selectivities of about 3 × 10^5^. PCE/N_2_ selectivities exceeding 10^7^ are predicted for EUO, MTW, and MOR, followed by IFR.

When looking at the pore system information listed in Table 2, it is noteworthy that the highest selectivities are predicted for frameworks possessing 1D pore systems of 12MR or 10MRs. A combination of 10 + 12MR pore openings does not appear to enhance the selectivity for any of the CEs studied. Regarding the numerical pore descriptors *d*(LIS), *d*(LDS), and *V*_occ_, there is no clear-cut correlation between any of these quantities and the CE/N_2_ selectivities shown in Figure 3. When considering that the pore size descriptors *d*(LIS) and *d*(LDS) are calculated for spherical probe molecules, whereas the CE molecules are distinctly nonspherical, this observation is hardly surprising. Nevertheless, it is worth pointing out that the zeolites that are predicted to be most selective toward CEs typically have pore systems characterized by a diameter of the largest included sphere that does not exceed 7.2 Å and a diameter of the largest diffusing sphere that falls between 5 and 6.5 Å. However, the reverse is not true; not all zeolites having *d*(LIS) and *d*(LDS) values in this range achieve high selectivities. As will be shown in more detail later, intricate features of the pore shape are responsible for the exceptionally high affinity of frameworks like EUO and MTW toward CEs.

Taking the full body of results compiled in Table S1.2 into consideration, the following additional observations may be useful for future work:

- Predicted selectivities for ETH over N_2_ are moderate, with the largest values on the order of 20 occurring for MTT, MTW, and EUO.
- The trends in CE/ETH selectivities are largely analogous to those presented above for CE/N_2_ mixtures.
- The EUO, TON, IFR, and MTW frameworks exhibit the highest selectivities for DCE isomers over nitrogen.
- Regarding differences in affinity toward the DCE isomers, the 10MR zeolites MFI, MTT, and especially TON exhibit the highest preference for *trans*-1,2-DCE over other isomers, with TON reaching *trans*-1,2-DCE/1,1-DCE and *trans*-1,2-DCE/*cis*-1,2-DCE selectivities of 7.0 and 3.1, respectively. Since the present work considered only those 10MR zeolites for which diffusion of larger CEs like PCE is possible, excluding several others like FER, MEL, and MWW, it seems worthwhile to perform a more extensive computational screening of 10MR zeolites when aiming to find optimized zeolite adsorbents for DCE isomer separation.

### CE Uptakes at Low Concentrations

**Figure 4:**
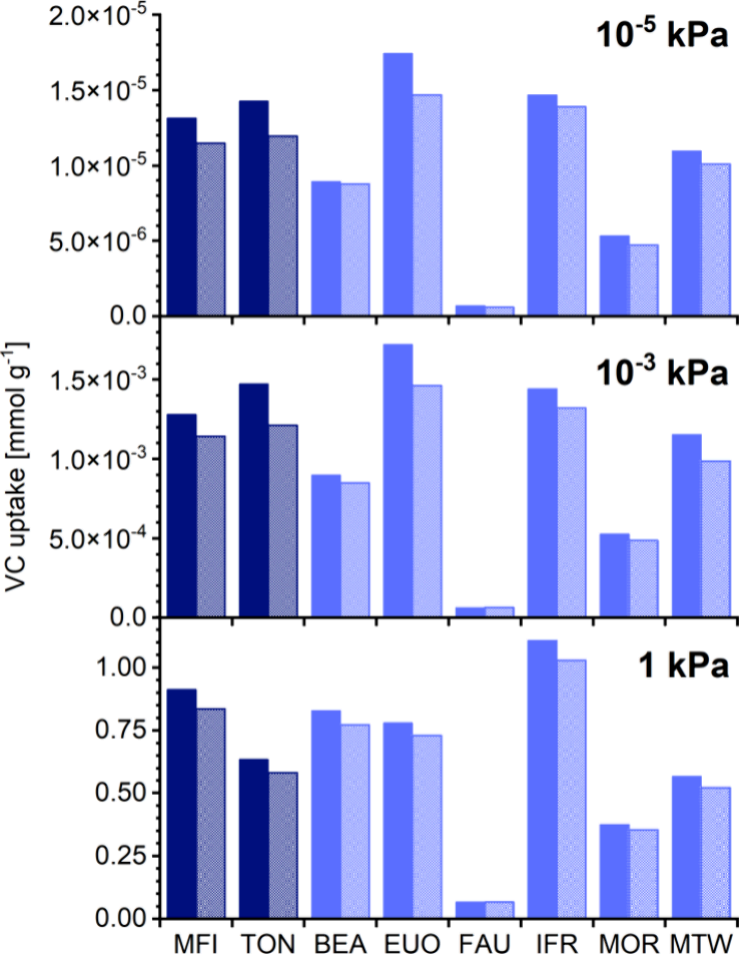
VC uptakes at different pressures obtained from VC adsorption simulations (solid columns) and VC/N_2_ mixture adsorption simulations (shaded columns).

As shown in the previous section, the CE/N_2_ selectivities in the limit of zero coverage become very large for large CEs such as TCE and PCE. However, as the target application is the removal of trace amounts, rather than bulk separation, these numbers give only limited insights into the potential usefulness of different zeolite frameworks for CE removal. In this regard, the uptake at low CE concentrations (= low fugacities) under ambient conditions is more relevant. To address this, adsorption isotherm simulations were performed for ETH and all CEs for a fugacity range from 10^–5^ to 1 kPa. In addition to computing single-component adsorption isotherms, simulations of VC/N_2_, TCE/N_2_, and PCE/N_2_ coadsorption were carried out, in which the fugacity of N_2_ was set to 101.325 kPa. Assuming ideal gas behavior, the partial pressures of the CEs in these mixtures correspond to concentrations ranging from 0.1 to 10,000 ppm (1 vol %). Full adsorption isotherms are shown in Tables S2.1 to S2.16. For the present discussion, uptakes at 10^–5^, 10^–3^, and 1 kPa are visualized in Figures 4 (VC) and 5 (TCE and PCE), focusing on the four commercially available zeolites and four frameworks exhibiting particularly high selectivities, namely, TON, EUO, IFR, and MTW.

**Figure 5:**
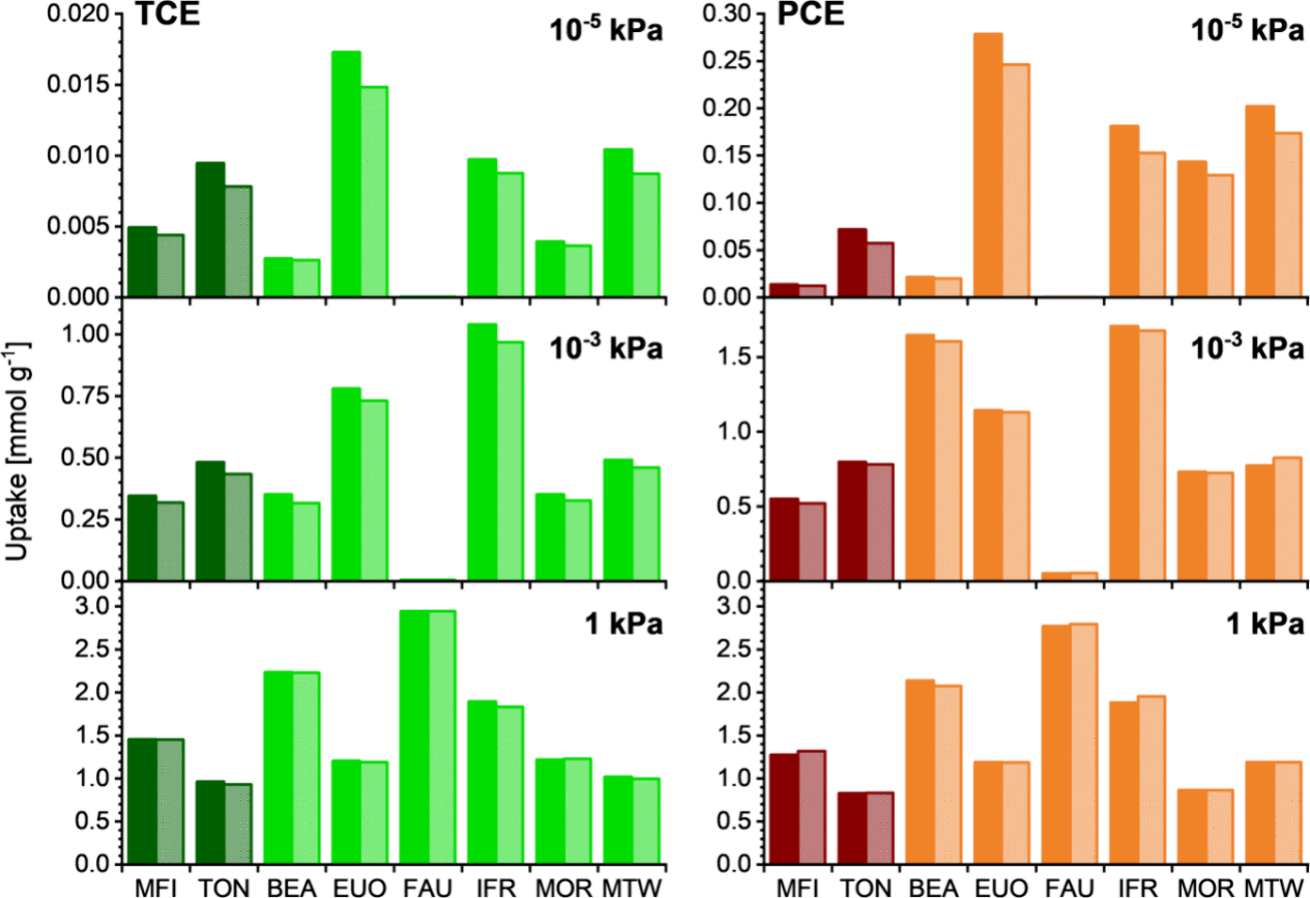
TCE and PCE uptakes at different pressures obtained from single-component adsorption simulations (solid columns) and CE/N_2_ mixture adsorption simulations (shaded columns).

The first thing that is worth noting when looking at the two figures is the close match of CE uptakes obtained from single-component adsorption simulations with those obtained from CE/N_2_ mixture simulations, with the reduction in CE uptake induced by N_2_ coadsorption never exceeding 20%. It can be inferred that the presence of nitrogen has only an insignificant impact on the attainable CE uptake and that single-component adsorption simulations should thus be sufficient for the systems and conditions considered here. With this in mind, the ETH and DCE isotherms that are reported in the Supporting Information, but not discussed in detail in this article, might be of use as starting points for future investigations.

As shown in Figure 4, EUO and IFR followed by TON and MFI take up the largest amounts of VC at 10^–5^ and 10^–3^ kPa. Even for these frameworks, however, the adsorbed amounts are very small, on the order of 10^–5^ and 10^–3^ mmol g^–1^, respectively, at the two pressures. This indicates that none of the zeolites are particularly attractive for the removal of VC in the ppm range. At 1 kPa, most of the zeolites adsorb significant amounts of VC, with the uptake of IFR exceeding 1 mmol g^–1^. However, such a high concentration of 1 mol % is not relevant for the removal of trace amounts.

Compared with VC, the uptakes of TCE and PCE computed for low pressures (Figure 5) are much higher. Although the trends for the two species are similar, the uptakes at 10^–5^ kPa are about 1 order of magnitude larger for PCE due to stronger host–guest interactions. EUO and MTW are predicted to adsorb the largest amounts of TCE and PCE at this pressure and should thus be best suited for their removal in the subppm range. Among the commercially available zeolites, MFI has the most beneficial properties for TCE removal, whereas MOR should perform better in PCE removal. At a pressure of 10^–3^ kPa, IFR exhibits the highest uptakes of both gases, which amount to 1.0 and 1.7 mmol g^–1^ for TCE and PCE, respectively. EUO constitutes the second-best adsorbent for TCE, whereas the commercially available BEA adsorbs almost as much PCE as IFR. The volume concentration of 10 ppm in the CE/N_2_ mixture is on the same order of magnitude as typical long-term exposure limits, indicating that these zeolites could find use for removal of traces of these contaminants, e.g., in a workplace context or in the treatment of exhaust gases. At 1 kPa, the amounts adsorbed in the different zeolites show a similar pattern for TCE and PCE. With few exceptions, the uptakes are well correlated with the accessible pore volume, *V*_acc_, indicating a complete filling of the pores by the guest molecules. This is corroborated by the adsorption isotherms visualized in the Supplementary EXCEL file, which show that saturation is essentially reached for all zeolites at 1 kPa, and, in many cases, at much lower pressures. For example, the PCE uptakes of EUO/IFR at 1 kPa are only 4%/10% higher than the respective uptakes at 10^–3^ kPa.

**Figure 6:**
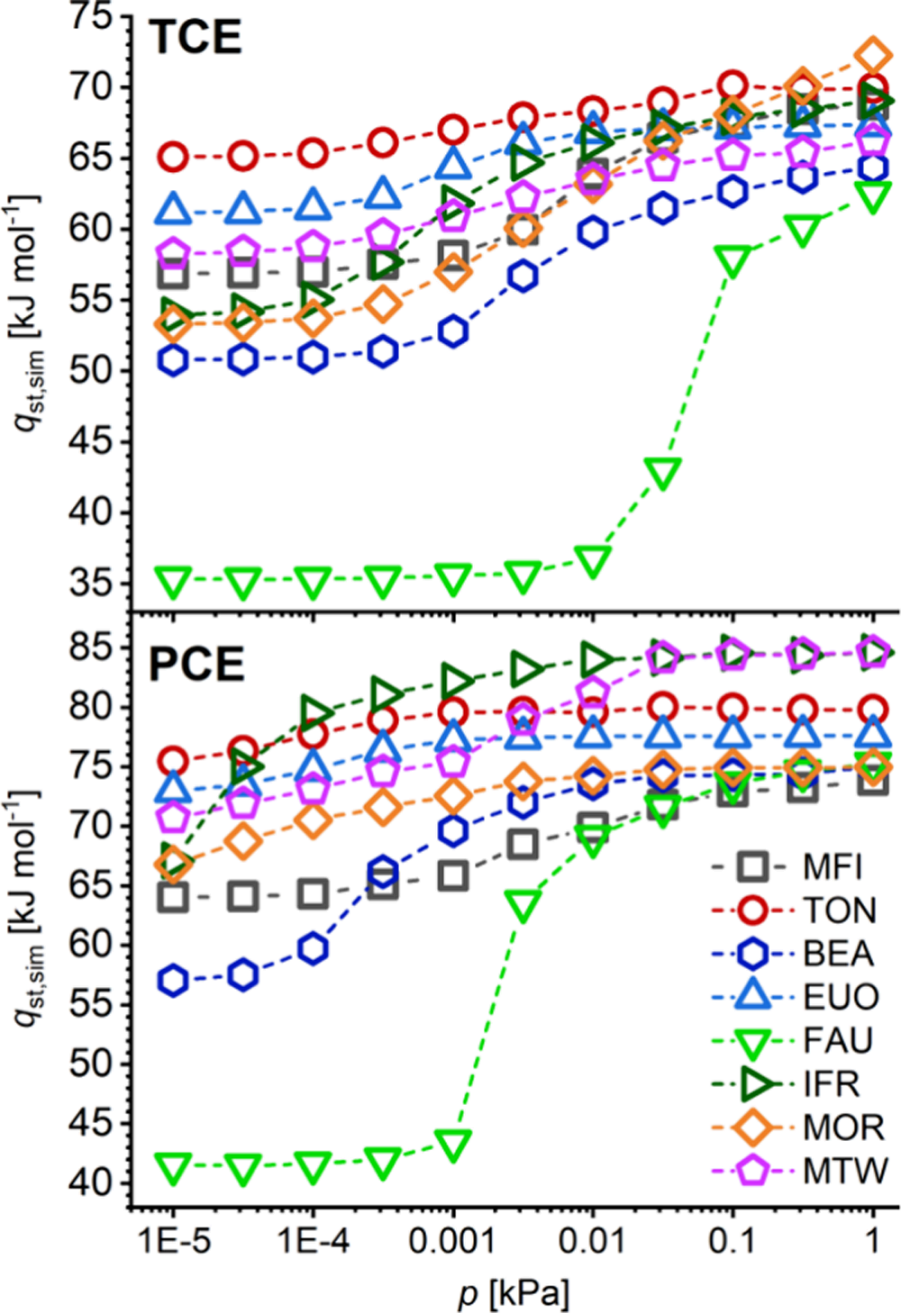
Isosteric heats of TCE and PCE adsorption.

The heats of TCE and PCE adsorption for the same eight zeolites are visualized in Figure 6. For ease of comparison with other figures, *q*_st_ is shown as a function of pressure, with pressure on a logarithmic scale; a visualization as a function of loading would deliver qualitatively analogous findings. Altogether, it is apparent that the affinity toward PCE is higher than toward TCE, and that the isosteric heats of adsorption increase with increasing loading. While the former observation is straightforwardly explained with the increased vdW interactions between PCE and the zeolites, the latter finding points to a prominent role in attractive guest–guest interactions. However, it is also noteworthy that both the quantitative differences among the *q*_st_ values obtained for TCE and PCE as well as the magnitude of the *q*_st_ increase with pressure vary considerably, depending on the framework type.

When one takes a closer look at Figure 6, the following aspects are noteworthy:

- TON and EUO exhibit the highest affinities toward both species at the lowest pressure, but the increases in *q*_st_ upon increasing pressure remain modest, on the order of 5 kJ mol^–1^. *q*_st_(PCE) is about 10 kJ mol^–1^ higher than *q*_st_(TCE) across the range of pressures.
- For MTW, the affinity toward both species at low pressures is also relatively high. While the increase of *q*_st_(TCE) with pressure is modest (as for TON and EUO), *q*_st_(PCE) increases more markedly, reaching almost 85 kJ mol^–1^ at 1 kPa.
- IFR shows a pronounced increase of the heats of adsorption with increasing pressure. Whereas the *q*_st_ values at 10^–5^ kPa are about 10 kJ mol^–1^ lower than those of TON, they increase by 15/17 kJ mol^–1^ for TCE/PCE, respectively. Together with MTW, IFR reaches the highest affinity toward PCE at 1 kPa.
- For MOR, a marked increase of *q*_st_(TCE) with pressure is observed, whereas the heat of PCE adsorption is much less affected. The difference between the two values at 1 kPa is unusually small, amounting to less than 3 kJ mol^–1^.
- For MFI, intermediate values of *q*_st_ are obtained for both species, and the increase with pressure is also moderate.
- For the remaining two systems, BEA and FAU, the heats of adsorption are rather low at low pressures, but increase significantly with increasing pressure. A qualitatively similar evolution has been observed experimentally for TCE in siliceous zeolite Y,^39^ although the overall increase was not quite as pronounced as predicted by the simulations reported here. In FAU, the large cages put few constraints on the position and orientation of the adsorbed molecules, allowing for local arrangements that maximize attractive guest–guest interactions.

### Simulation Snapshots

**Figure 7:**
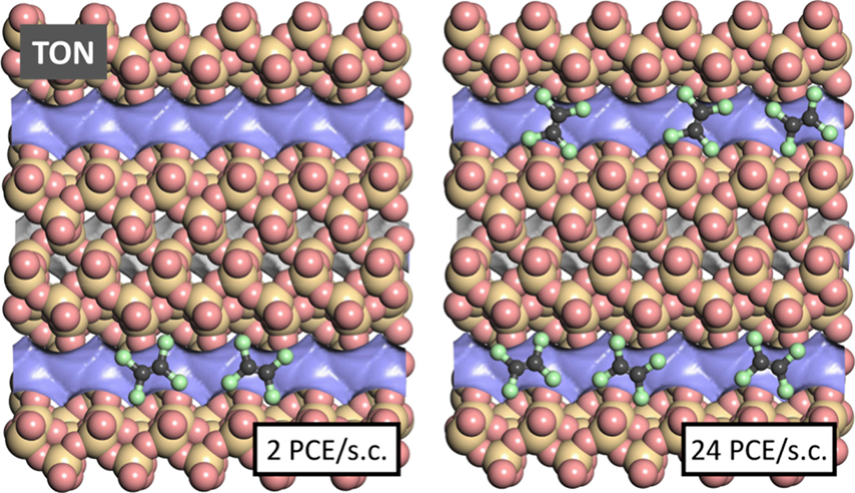
Representative snapshots from fixed-loading simulations of PCE in TON. Because the simulation cell (s.c.) contains channels that are not included in the shown portion of the structure, not all molecules are necessarily visible in this and the following figures.

**Figure 8:**
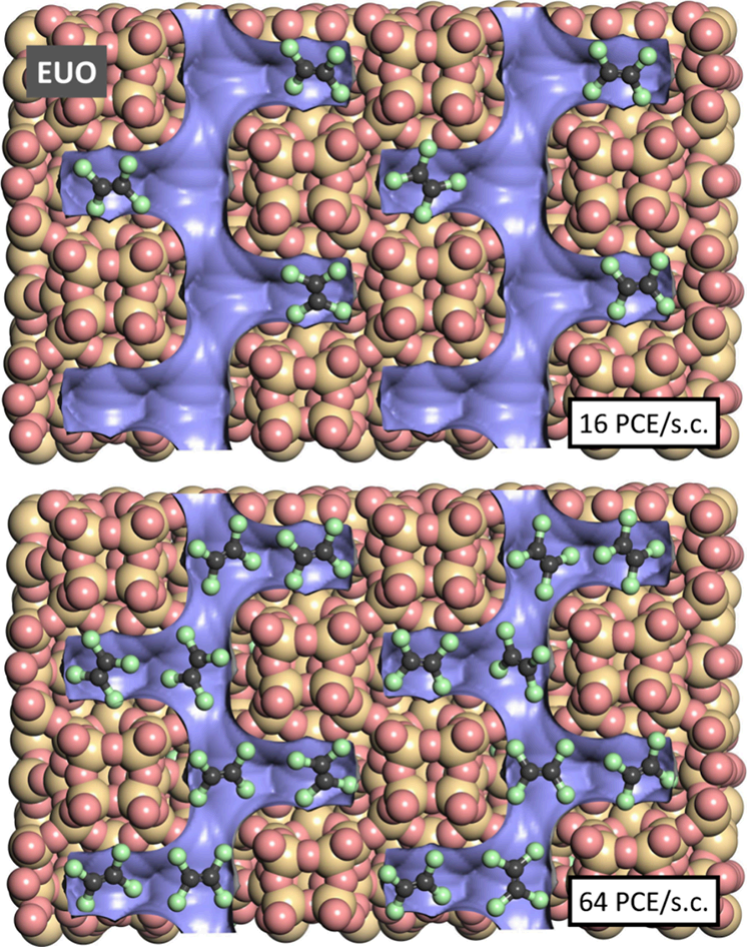
Representative snapshots from fixed-load simulations of PCE in EUO.

Altogether, it is apparent that the framework topology affects not only the heat of adsorption in the limit of low coverage but also the extent of guest–guest interactions, including – in some cases – intricate differences between TCE and PCE. In order to develop a more detailed understanding of the adsorption behavior in zeolites of particular interest, fixed-loading MC simulations were performed for one or two selected loadings, depending on the system, and representative simulation snapshots were visualized. Figures 7 and 8 show such snapshots for TON and EUO for two PCE loadings that correspond approximately to the uptakes at 10^–5^ kPa (TON: 2 PCE per simulation cell [s.c.], EUO: 16 PCE/s.c.) and at saturation, which is almost reached at 10^–3^ kPa (TON/EUO: 24/64 PCE/s.c.). The snapshots obtained for lower loadings show that PCE fits very nicely into the zigzag channels of TON and into the side pockets of the EUO channels, with the local environments allowing short contacts of all 4 Cl atoms to framework atoms. This tight fit explains the strong host–guest interactions that are responsible for the high *q*_st_(PCE) at low pressures. Under near-saturation conditions, the channels of TON are gradually filled, whereas additional molecules in EUO are adsorbed in the main channels. In both zeolites, the adsorbed molecules lie more or less in the same plane, with Cl atoms of adjacent molecules pointing toward each other. While the guest–guest interactions felt by an individual molecule will depend on the specific local arrangement, it can be inferred that attractive vdW interactions are at least partially counterbalanced by repulsive electrostatic interactions between negatively polarized Cl atoms, especially at high guest loadings. Therefore, the overall contribution of guest–guest interactions is only slightly attractive, resulting in a modest increase of *q*_*st*_(PCE) with pressure.

**Figure 9:**
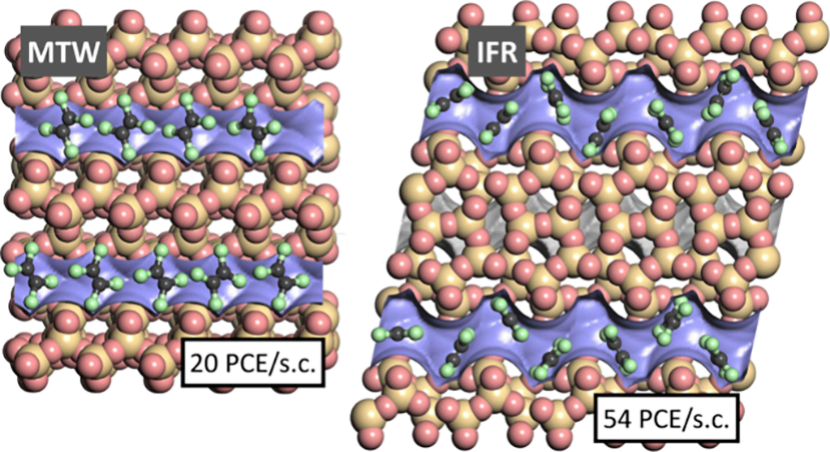
Representative snapshots from fixed-loading simulations of PCE in MTW and IFR.

For MTW, the simulation snapshot obtained for near-saturation conditions (20 PCE/sc), visualized in the left panel of Figure 9, shows that the PCE molecules fit very well into the pores of this system. Similar to the observations made for TON, the Cl atoms point into “bulges” of the undulating channels, resulting in strong host–guest interactions. Unlike in TON, however, the adsorbed PCE molecules do not lie in the same plane but assume a preferred orientation that is somewhat tilted with respect to the running direction of the channels. This tilted arrangement allows for stronger attractive guest–guest interactions, for example, through electrostatic interactions between Cl atoms and positively polarized C atoms of adjacent PCE molecules. In IFR, visualized in the right panel of Figure 9, yet another scenario is observed: Since the channels of this zeolite are somewhat wider, the PCE molecules are displaced from the center of the zigzag channels, mostly interacting with one side of the pore wall. This less-than-optimal fit explains why the heat of adsorption in IFR at low coverages is much lower than in the zeolites discussed so far. The strong guest–guest interactions that are responsible for the large increase of *q*_st_(PCE) with loading can also be understood on the basis of the snapshot: Across some parts of the channels that are visible in the figure, the PCE molecules form “chains” in which two chlorine atoms of one molecule point toward the central, positively polarized area of one neighboring molecule. Moreover, the intramolecular Cl···C distances are often close to the sum of the vdW radii.

**Figure 10:**
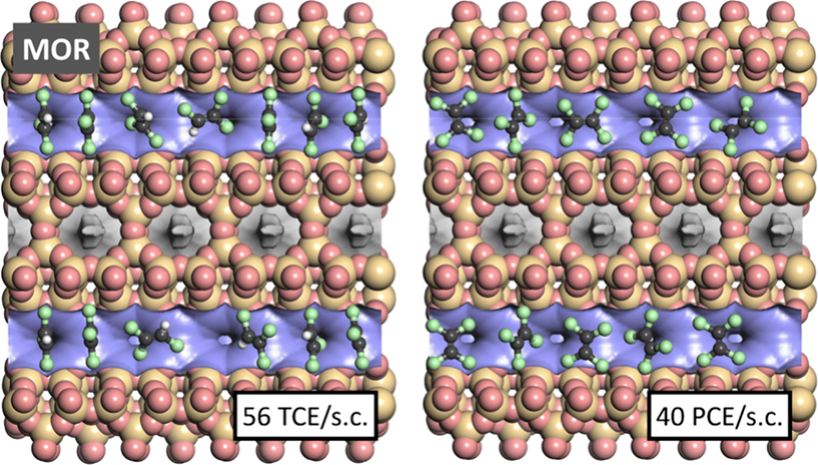
Representative snapshots from fixed-load simulations of TCE and PCE in MOR.

Finally, it is worth taking a look at MOR, where rather different trends in affinity were observed for PCE and TCE. A snapshot computed for high TCE loadings, visualized in the left panel of Figure 10, shows that the majority of adsorbed molecules are oriented essentially perpendicular to the running direction of the channels. Across large portions of the channel, the stacking of the TCE molecules is largely commensurate with the slight undulation of the MOR channels. In contrast, adsorbed PCE molecules are never found in such a perpendicular orientation, but they always lie more or less in the plane of the elliptical 12MR channels (right panel of Figure 10). Apparently, PCE, although being only slightly larger than TCE, is too bulky to assume an orientation perpendicular to the channel axis, resulting in less efficient packing of the adsorbed molecules. For this reason, the attainable PCE loading is significantly smaller (0.9 mmol g^–1^, compared to 1.2 mmol g^–1^ for TCE), and the contribution of attractive guest–guest interactions is also reduced considerably.

## Conclusions

Molecular simulations were carried out to study the adsorption of CEs and ETH in a structurally diverse set of all-silica zeolites, with the discussion of the results focusing on VC and, most prominently, TCE and PCE. The Henry constant simulations performed in the first part of the study showed wide variations in affinity, highlighting the important influence of pore topology on the interaction with CEs. Due to the limited computational expense, such simulations could straightforwardly be extended to include other zeolite frameworks and/or other (emerging) environmental contaminants, such as fluorinated hydrocarbons, 1,3-butadiene, acrylonitrile, ethylene oxide, or others. Naturally, a key prerequisite for such extensions is the availability of sufficiently accurate force field parameters, whose suitability should be ascertained through validation against experimental data whenever possible.

The following GCMC simulations, which covered a wide range of pressures, showed that VC uptakes at parts per million concentrations are too low to be practically relevant for VC removal. In contrast, several zeolites showed substantial uptakes of TCE and PCE in the range of 0.1 to 10 ppm, indicating that they might be suitable adsorbents for the removal of trace amounts of these species. Among the frameworks studied, the 12MR zeolites EUO, IFR, and MTW would be of most interest for future experimental studies; however, these zeolites are, to the author’s knowledge, not commercially available. Focusing on those zeolites that are actually marketed in high-silica form, BEA (available as zeolite beta, which shows stacking disorder of the beta layers^31^) and MOR (mordenite) appear as most promising choices. This finding is particularly relevant when considering that most previous experimental studies of CE adsorption concentrated on MFI- and FAU-type zeolites. As shown for selected cases, a detailed analysis of simulation snapshots can provide insights into the role of host–guest and guest–guest interactions. Such insights can help to develop descriptors that may facilitate the identification of other zeolites (or related crystalline microporous materials) that are of particular interest as adsorbents for CE removal. These could then be prioritized in future work.

It should be noted that GCMC simulations including the coadsorption of N_2_ indicate that its presence does not have a major impact on CE uptake. Real-world scenarios, however, would typically involve the adsorption of CEs from complex gas mixtures, where competitive adsorption of other species is likely to affect the attainable CE adsorption capacities. Such issues, which will depend on the specific composition of the gas feed in a given setting, should be addressed in future experimental and computational studies. One aspect that is of particular importance is the coadsorption of water, which is ubiquitous and often strongly adsorbed due to its large dipole moment. While perfect all-silica zeolites are hydrophobic, the presence of defects tends to increase the hydrophilicity, thus promoting the competitive adsorption of water. Inclusion of such defects and of coadsorbed water molecules would constitute an interesting task for future molecular simulation studies. Additional challenges arise when considering the adsorption from an aqueous solution, rather than from the gas phase, in molecular simulations. In a recent study focusing on 1,4-dioxane adsorption in zeolites, a gauge cell MC approach was employed to model the adsorption from aqueous solution in the parts per billion (ppb) range.^81^ It could be very interesting to exploit this approach in simulations of CE adsorption from water.

In terms of diffusion properties, only a qualitative preliminary assessment was made in the context of the present study. According to this assessment, diffusion limitations should not play a major role, especially in zeolites with 12MR pore openings. However, numerical modeling of the diffusion properties could be of interest when targeting the application of zeolite membranes for CE removal, where the permeation selectivity of the membrane can be expressed as a product of the adsorption and diffusion selectivities.^82^

Another aspect that has not been the focus here is the regeneration of the zeolite adsorbent. In view of the high thermal stability of high-silica zeolites, which typically exceeds 1000 °C, thermal regeneration appears as a straightforward option. Beyond that, doping with catalytically active metals could constitute a pathway toward CE decomposition that requires less thermal energy. In such systems, the interplay of adsorption properties (enabling efficient CE capture) and catalytic properties (affording complete CE decomposition) should be optimized in order to maximize the CE removal efficiency.

## Data Availability

ZIP archive Guest_molecules_CAR.zip containing molecular structures of guest molecules (in CAR format) and ZIP archive Zeolites_GULP_opti_CIFs.zip containing GULP-optimised zeolite structures (in CIF format) are available from ChemRXiv: https://doi.org/10.26434/chemrxiv-2024-w6kxl.

## Supporting Information Available

Table SI (zeolite structures) and SII (force field parameters) (PDF)
Full results of Henry constant simulations (Table S1.1), selectivities (Table S1.2), and GCMC simulations (Table S2.1 to S2.16) (XLSX)
