# Supplementary material for: Molecular Simulation Study of All-Silica Zeolites for the Adsorptive Removal of Airborne Chloroethenes
Source: Langmuir. 2025 Jan 6;41(2):1344–55. doi: 10.1021/acs.langmuir.4c03947 (PMC11755781; doi:10.1021/acs.langmuir.4c03947)
Supplement: Supplementary file 1 — la4c03947_si_001.pdf [file la4c03947_si_001.pdf]

Molecular simulation study of all-silica zeolites for the adsorptive removal of airborne chloroethenes

Michael Fischer[a,b*]

[a] *Faculty of Geosciences, University of Bremen, Klagenfurter Straße 2-4, 28359 Bremen, Germany*

[b] *Bremen Center for Computational Materials Science (BCCMS) and MAPEX Center for Materials and Processes, University of Bremen, 28359 Bremen, Germany*

* E-mail: michael.fischer@uni-bremen.de

SUPPORTING INFORMATION PDF

Table SI: Space group symmetry and cell parameters of GULP-optimised zeolite structures and cell multiplications employed in Henry constant calculations and GCMC simulations. Zeolites with FTCs highlighted in bold were included in the main calculations reported in the article, the remaining ones were used only in auxiliary or preliminary calculations.

**FTC**	**Space group (ITA no.)**	***a*, *b*, *c* [Å]**	***α*, *β*, *γ* [°]**	**Cell (Henry)**	**Cell (GCMC)**
CHA	$R\bar{3}m$ (166)	13.537, 13.537, 14.553	90, 90, 120	2×2×2	n/a
FER	$Pnnm$ (58)	14.000, 7.393, 18.581	90, 90, 90	2×3×1	n/a
MEL	$P\bar{4}n2$ (118)	19.882, 19.882, 13.336	90, 90, 90	1×1×2	n/a
**MFI**	$P2_1/n$ (14)	19.740, 19.980, 13.320	90, 90.81, 90	1×1×2	2×2×2
**MTT**	$P2_1$ (4)	22.235, 5.004, 21.608	90, 90.03, 90	1×4×1	2×6×2
MWW	$P622$ (177)	14.174, 14.174, 24.964	90, 90, 120	2×2×1	n/a
**TON**	$Pbc2_1$ (29)	13.815, 17.388, 5.002	90, 90, 90	2×2×5	2×2×5
**TUN**	$C2/m$ (12)	28.084, 19.828, 19.397	90, 92.22, 90	1×1×1	1×2×2
**AFI**	$P3_1$ (144)	13.597, 13.597, 24.904	90, 90, 120	2×2×1	2×2×1
**BEA**	$P4_122$ (91)	12.464, 12.464, 26.225	90, 90, 90	2×2×1	2×2×1
**EUO**	$Pba2$ (32)	13.633, 22.129, 20.064	90, 90, 90	2×1×1	2×2×2
**FAU**	$Fd\bar{3}m$ (227)	24.227, 24.227, 24.227	90, 90, 90	1×1×1	1×1×1
**IFR**	$C2/m$ (12)	18.604, 13.422, 7.633	90, 77.97, 90	1×2×3	2×2×4
**MEI**	$P6_3/m$ (176)	13.036, 13.036, 15.592	90, 90, 120	2×2×2	2×2×2
**MOR**	$P2_12_12_1$ (19)	18.023, 20.042, 7.432	90, 90, 90	1×1×4	2×2×4
**MTW**	$C2/c$ (15)	24.969, 5.016, 24.178	90, 107.16, 90	1×4×1	1×5×1
**IWR**	$Pmmn$ (59)	20.983, 13.437, 12.489	90, 90, 90	1×2×2	2×2×2
**MSE**	$P\bar{1}$ (2)	18.232, 18.244, 20.029	89.65, 90.27, 89.92	1×1×1	2×2×2
**CFI**	$P1$ (1)	13.630, 5.049, 25.597	90.29, 89.91, 90.43	2×4×1	2×5×1
**DON**	$Pcmn$ (62)	18.916, 8.469, 22.992	90, 90, 90	2×3×1	2×3×2

Table SII: Atom types and non-bonded parameters used in force field calculations. Partial charges q and Lennard-Jones parameters R_0 and D_0 are given for each atom type. Note that all parameters are intended for use with a 9-6 LJ potential. NI means no LJ interaction.

Description	Atom type	q [e]	R_0 [Å]	D_0 [kcal mol^{-1}]
(C=)C, bonded to 2 H atoms	c=	−0.2536	3.90	0.064
(C=)C, bonded to 1 H and 1 Cl	c=	−0.0248	3.90	0.064
(C=)C, bonded to 2 Cl atoms	c=	0.204	3.90	0.064
H, bonded to C(=C)	hc	0.1268	NI	NI
Cl, bonded to C(=C)	cl	−0.102	3.92	0.2247
N in N_2	nz	0	3.8008	0.0598
Si in zeolite (PCFF)	sz	0.5236	NI	NI
O in zeolite (PCFF)	oss	−0.2618	3.4506	0.1622
Si in zeolite (PCFF/Emami)	sz	1.100	4.20	0.080
O in zeolite (PCFF/Emami)	oss	−0.550	3.60	0.040
